# Comment on "The role of wildlife (wild birds) in the global transmission of antimicrobial resistance genes"

**DOI:** 10.24272/j.issn.2095-8137.2017.023

**Published:** 2017-07-18

**Authors:** Mashkoor Mohsin, Shahbaz Raza

**Affiliations:** Institute of Microbiology, University of Agriculture, Faisalabad, Pakistan

## Dear Editor,

We read with interest the article by Wang and colleagues regarding the role of wildlife in the transmission of antimicrobial resistance (AMR) (Wang et al., 2017). Although we appreciate the efforts in reviewing this important topic, we would like to comment on some statements that we believe are not up-to-date or properly cited.

The authors mentioned only two reports of *Escherichia coli* carrying plasmid-mediated colistin resistance gene *mcr-1* in wild birds in their review. The first report was on European herring gulls from Lithuania (Ruzauskas & Vaskeviciute, 2016) and the second was on Kelp gulls (*Larus dominicanus*) from Argentina (Liakopoulos et al., 2016). In our 2016 article, we already reported, for the first time, on the plasmid-mediated colistin resistance extended-spectrum β-lactamase-producing *E. coli* strain PK-13 from a wild migratory bird (Eurasian coot, *Fulica atra*) in Asia (Mohsin et al., 2016). However, the authors have not described our findings in their review. Furthermore, it is important to note that the *E. coli* strain PK-13 carries the IncI2 plasmid, which is in agreement with the original Chinese study (Liu et al., 2016) and previous reports from wild birds (Ruzauskas & Vaskeviciute, 2016; Liakopoulos et al., 2016). Therefore, it is likely that plasmid IncI2 is involved in the spread of the *mcr-1* gene in *E. coli* isolates from wild birds. Every winter, Pakistan hosts more than a million wild migratory birds from Siberia and Central Asia (Mohsin et al., 2016). There is already a dearth of data on the presence of *mcr-1* in wild birds and omitting the only article from Asia is misleading and does not provide up-to-date information to the reader. We also recently reported on the high carriage of CTX-M-15-producing *Klebsiella* *pneumoniae* in wild migratory birds in Pakistan (Raza et al., 2017). Finally, we agree with the authors that long-range migration of birds could be involved in the global dissemination of AMR.
